# Bone-Specific Alkaline Phosphatase as a Complementary Diagnostic Marker for the Assessment of Children and Adolescents with Secondary Osteoporosis

**DOI:** 10.3390/diagnostics15050630

**Published:** 2025-03-05

**Authors:** Eunha Bae, Soo Yeun Sim, Su Jin Park, Sung Eun Kim, Seulki Kim, Shin-Hee Kim, Won Kyoung Cho, Kyoung Soon Cho, Min Ho Jung, Byung-Kyu Suh, Moon Bae Ahn

**Affiliations:** Department of Pediatrics, College of Medicine, The Catholic University of Korea, Seoul 06591, Republic of Korea; becca.eunha@gmail.com (E.B.); sooyeunsim8@gmail.com (S.Y.S.); jetaime_7@naver.com (S.J.P.); libaigh2@naver.com (S.E.K.); seulki12633@gmail.com (S.K.); tigger1018@naver.com (S.-H.K.); wendy626@catholic.ac.kr (W.K.C.); soon926@hanmail.net (K.S.C.); jmhpe@catholic.ac.kr (M.H.J.); suhbk@catholic.ac.kr (B.-K.S.)

**Keywords:** bone turnover markers, bone-specific alkaline phosphatase, children, adolescents, secondary osteoporosis, lumbar spine bone mineral density

## Abstract

**Background/Objective**: With increasing cases of osteoporosis in children and adolescents, the need for timely diagnosis, management, and follow-up has become important. This study aimed to determine whether bone turnover markers (BTMs), particularly serum bone-specific alkaline phosphatase (BsALP) and serum C-telopeptide of collagen type 1 (CTx), accurately reflect BMD. **Methods**: In this retrospective study, 280 post-puberty males and females who were previously diagnosed with hemato-oncologic, rheumatic, gastrointestinal, and endocrinologic diseases at a single tertiary care center were reviewed. The association between the lumbar spine bone mineral density (LSBMD) Z-scores and BTMs, such as BsALP and CTx, were assessed. The LSBMD was measured in the anterior–posterior direction using DXA, and BTMs were determined using the blood samples obtained. **Results**: Of the 280 patients, 95 were male (33.9%), and the mean age was 15.4 ± 2.07 years. With multivariate regression analysis, LSBMD Z-scores and BsALP showed a negative correlation with *p* < 0.007, while CTx was not statistically significant. The logistic regression models showed that after adjusting for underlying diseases and sex, as BsALP increased, the probability of LSBMD Z-score being ≤−2 increased with an odds ratio of 1.043 (*p* = 0.048). When comparing BTMs with vertebral fracture while adjusting for underlying diseases and sex, as BsALP increased, the probability of vertebral fracture increased with an odds ratio of 1.035 (*p* = 0.005). **Conclusions**: The positive correlation between BsALP and LSBMD Z-scores being ≤−2, as well as with vertebral fracture after adjusting for underlying diseases and sex, suggests the possible application of BsALP as a predictor of bone health in patients.

## 1. Introduction

Children and adolescents with chronic diseases may experience various endocrine complications, not only because of the natural course of the disease but also due to the various side effects of the treatments they receive. Among these endocrine complications, low bone mass and/or vertebral fractures are common and should not be underestimated or neglected, as bone health is closely related to the quality of life of patients [1].

Osteoporosis is a systemic skeletal disease characterized by a deterioration of the microarchitecture of the bone tissue that leads to low bone mineral density (BMD) and an increased risk of bone fracture [2]. It is mainly recognized as a disease in adulthood; however, an increasing number of children and adolescents are affected as well [3]. Childhood and adolescence are an important stage for bone mineral development, with peak bone mass reached by the age of 20 [4]. Unlike in adults, children and adolescents with low bone mass should be carefully examined to identify the secondary causes. If no such cause is identified, low bone mass may be the primary cause and possibly related to rare gene variants [5]. Children and adolescents with chronic underlying diseases such as acute leukemia may experience a decrease in BMD due to the infiltration of leukemic cells in the bone, as well as the use of long-term immunosuppressive drugs and/or organ transplantation [6]. Additionally, diseases such as rheumatic and inflammatory bowel diseases are associated with inflammatory cytokines, which can decrease bone formation, enhance bone resorption, and ultimately damage bone health [7].

In adults, the gold standard for diagnosing and evaluating osteoporosis is BMD analysis, which is measured by dual-energy X-ray absorptiometry (DXA) based on the World Health Organization (WHO) criteria [8]. DXA is the method which is generally used to measure BMD in postmenopausal women and men over the age of 50 years. Osteoporosis in adults is defined as the T-value of either the lumbar spine or hip joint BMD being <−2.5 [9]. On the contrary, pediatric osteoporosis is defined as having one or more vertebral compression fractures without local disease or high-energy trauma, such as road traffic accidents or falls from above 3 m, according to the guidelines of the International Society of Clinical Densitometry [10]. The preferable sites for assessing BMD in pediatrics are the posteroanterior lumbar spine and total body less head [11]. Specifically, patients with both BMD Z-scores being <−2.0 measured by DXA of the hip and/or lumbar spine and a significant fracture history of two or more long bone fractures by the age of 10 or ≥3 by the age of 18 are considered to have osteoporosis [12]. However, the value of BMD as a diagnostic tool in pediatric patients is somewhat limited because of several factors. First, the relationship between fracture risk and BMD Z-score is not well-known in children and adolescents. Second, relatively short stature and/or delayed skeletal development in some children may lead to an underestimation of the BMD Z-score. Finally, patients with BMD Z-scores > −2.0 can also have vertebral fractures [13]. Thus, a complementary diagnostic tool to better predict the risk of osteoporosis in patients to identify those with a higher probability of fracture before it actually occurs would be useful.

Osteoporosis is characterized by bone fragility, which is multifactorial, including bone microarchitecture, microdamage, and bone remodeling rate [14]. Of these, the bone remodeling rate, which is affected by bone resorption and formation, can be evaluated by various bone turnover markers (BTMs) [15]. Among various BTMs, serum bone-specific alkaline phosphatase (BsALP), osteocalcin (OC), and procollagen type 1 N-terminal propeptide (P1NP) represent bone formation, while carboxyl-terminal crosslinked telopeptide of type 1 collagen (CTx) and amino-terminal crosslinked telopeptides of type 1 collagen (NTx) represent bone resorption [16]. These BTMs, when combined with BMD analysis, may be useful for early detection of osteoporosis.

In adults, the use of BTMs to monitor anti-osteoporotic treatments has been studied and is recommended [17]. However, how BTMs can be used in children and adolescents with osteoporosis is not fully known, as well as their possible use as diagnostic criteria and/or as predictors of fracture risk. This study aimed to determine whether BTM accurately represents BMD. Of many BTMs, serum BsALP, which reflects bone formation, and serum CTx, which reflects bone resorption, were studied.

## 2. Materials and Methods

### 2.1. Participants

Pediatric patients who were previously diagnosed with various chronic diseases and were evaluated for their bone health were included in this study. The chronic diseases were categorized into hemato-oncologic, rheumatic, gastrointestinal, and endocrinologic diseases at a single tertiary care center. Post-puberty age males and females with Tanner stage ≥ II were included. Patients who had received DXA 6 months or more apart from when BTM sampling was performed were excluded. Patients with primary bone disease or who underwent ongoing therapeutic intervention that could affect bone density were also excluded. Patients in the miscellaneous category included patients with anorexia nervosa, syndromic diseases, nephrotic syndrome, adrenal cortical adenoma, and neurofibromatosis.

This retrospective cross-sectional study was approved by the Institutional Review Board of the Catholic University of Korea (KC24RISI0359) and was conducted in accordance with the principles of the Declaration of Helsinki. The requirement for informed patient consent was waived as no interventions or further examinations were performed.

### 2.2. Disease Category

#### 2.2.1. Hemato-Oncologic Disease

Patients who had been previously diagnosed with leukemia, lymphoma, histiocytosis, solid organ tumor, or bone marrow failure, determined by bone marrow biopsy or radiological findings, were included. They received treatment, including multidrug chemotherapy, focal or total body irradiation, repeated blood transfusions, and surgery, with or without peripheral blood stem cell transplantation. The treatment was carried out according to the standardized protocol of the Division of Pediatric Hemato-Oncology of our institution.

#### 2.2.2. Rheumatic Disease

Patients who had been previously diagnosed with systemic lupus erythematosus, juvenile rheumatic arthritis or idiopathic arthritis, Sjogren–Larsson syndrome, Behçet’s disease, and juvenile dermatomyositis, as indicated by the presence of disease-specific antibodies, were included. They were treated with glucocorticoids, nonsteroidal anti-inflammatory drugs, and disease-modifying antirheumatic drugs. The treatment plan followed the standardized protocol of the Division of Pediatric Rheumatology of our institution.

#### 2.2.3. Gastrointestinal Disease

Patients who had been previously diagnosed with inflammatory bowel disease, including Crohn’s disease and ulcerative colitis, based on gastroduodenocolonoscopic and radiological findings, were included. They were treated with glucocorticoids, 5-aminosalicylic acids, and immune modulators (methotrexate or azathioprine), with or without biologics (such as antitumor necrosis factor or anti-integrin). The treatment was carried out according to the standardized protocol of the Division of Pediatric Gastroenterology and Nutrition of our institution.

#### 2.2.4. Endocrine Disease

Patients who had been previously diagnosed with growth hormone deficiency, idiopathic short stature, constitutional delay in growth and puberty, hyperthyroidism, hypothyroidism, idiopathic osteoporosis, or congenital adrenal hyperplasia were included. Some of them received hormone replacement therapy. Patients with primary bone disease or with diseases that could affect bone density were excluded. The treatment was carried out according to the standardized protocol of the Division of Pediatric Endocrinology of our institution.

### 2.3. Data Collection

#### 2.3.1. Anthropometric Measurements

Height (cm) was measured using a Harpenden Stadiometer (Holtain^®^, Crymych, UK), while weight (kg) was measured using a Simple Weighing Scale (CAS^®^, Seoul, Republic of Korea). The body mass index (BMI) (kg/m^2^) was calculated and converted to age- and sex-matched standard deviation (Z)-scores based on the national growth chart.

#### 2.3.2. BMD Assessment

The BMDs of the lumbar spine from L1 to L4 (LSBMD) and right and left femur were measured in the anterior–posterior direction using DXA (HorizonW DXA system^®^, Hologic Corp., Marlborough, MA, USA), whereas the age- and sex-matched Z-scores for areal BMD (g/cm^2^) were determined based on the native Normative Pediatric reference data. All DXA measurements were performed by a single radiographer who was blinded to the clinical history of the patients.

#### 2.3.3. Serum BTMs

Serum BTMs analyzed in this study included BsALP (DXI-800, Beckman Coulter, Brea, CA, USA), CTx (Cobas e801, Roche, Basal, Switzerland), P1NP (Cobas e801, Roche, Basal, Switzerland), calcium (Cobas c702, Roche, Basal, Switzerland), phosphorus (Cobas c702, Roche, Basal, Switzerland), 25-hydroxycholecalciferol vitamin D total level (25OHD3) (Atellica IM 1600, Siemens, Munich, Germany), and intact parathyroid hormone (PTH) (Cobas e801, Roche, Basal, Switzerland). They were determined using the blood sample obtained less than 6 months apart from when BMD was measured. The patients were told to perform the blood sampling early in the morning after fasting for more than 8 h to control any errors caused by circadian variation or blood sugar level.

### 2.4. Statistical Analysis

For all descriptive variables, the normality of distribution was determined using the Shapiro−Wilk test. When comparing two groups of LSBMD Z-scores with each BTM, the Mann−Whitney U test was used. The same test was used to compare the vertebral fracture with BTMs. Spearman’s rho (ρ) was calculated to describe the correlation between BsALP with other BTMs such as calcium, phosphorous, 25OHD3, and PTH, as well as with CTx. Univariate and multivariate regression were performed to estimate the beta coefficients (β) for factors associated with the LSBMD Z-scores. Subsequently, a multiple logistic regression analysis of BMI Z-scores, age when the sampling was performed, BTMs including BsALP, calcium, phosphorus, and 25OHD3 with LSBMD Z-scores as dependent variables was performed to calculate the odds ratios (OR) after adjusting for the underlying diseases and sex. The chi-square test was performed to find the correlation between the prevalence of vertebral fractures and the degree of low bone mass. All statistical analyses were performed using SPSS software (version 24.0; IBM Corp.^®^, Armonk, NY, USA).

## 3. Results

### 3.1. Demographics and Clinical Characteristics

The demographics of the study population are shown in Table 1. In this retrospective study, 280 post-pubertal males aged 12.5–18 years and females aged 10.5–18 years were assessed. Of the 280 patients, 95 were male (33.9%), and the mean age was 15.4 ± 2.07 years old. Among the study population, 61.4% were diagnosed with hemato-oncologic diseases, 12.9% with rheumatic diseases, 12.5% with gastrointestinal diseases, 5.4% with endocrinology, and 7.9% with miscellaneous diseases. Miscellaneous included ten patients with anorexia nervosa, eight with syndromic diseases, two with nephrotic syndrome, one with adrenal cortical adenoma, and one with neurofibromatosis. Among hemato-oncologic patients, the most prevailing disease diagnosed was leukemia, with 126 cases either originating from the lymphoid or myeloid white blood cells, followed by 23 patients with primary bone marrow failure, such as aplastic anemia. The most commonly diagnosed disease for rheumatic patients was systemic lupus erythematosus with 22 patients, and for gastrointestinal patients was Crohn’s disease with 25 patients. The mean LSBMD Z-score was −0.52 ± 1.23, with 41.4% of the patients showing a Z-score between −1.0 and 0, 21.1% between −2.0 and −1.0, 9.3% between −3.0 and −2.0, and 2.5% being ≤−3.0. Among the included patients, 79 patients (28.2%) had vertebral fractures.

### 3.2. Relationship Between Low Bone Mass and BTMs

**Figure 1:**
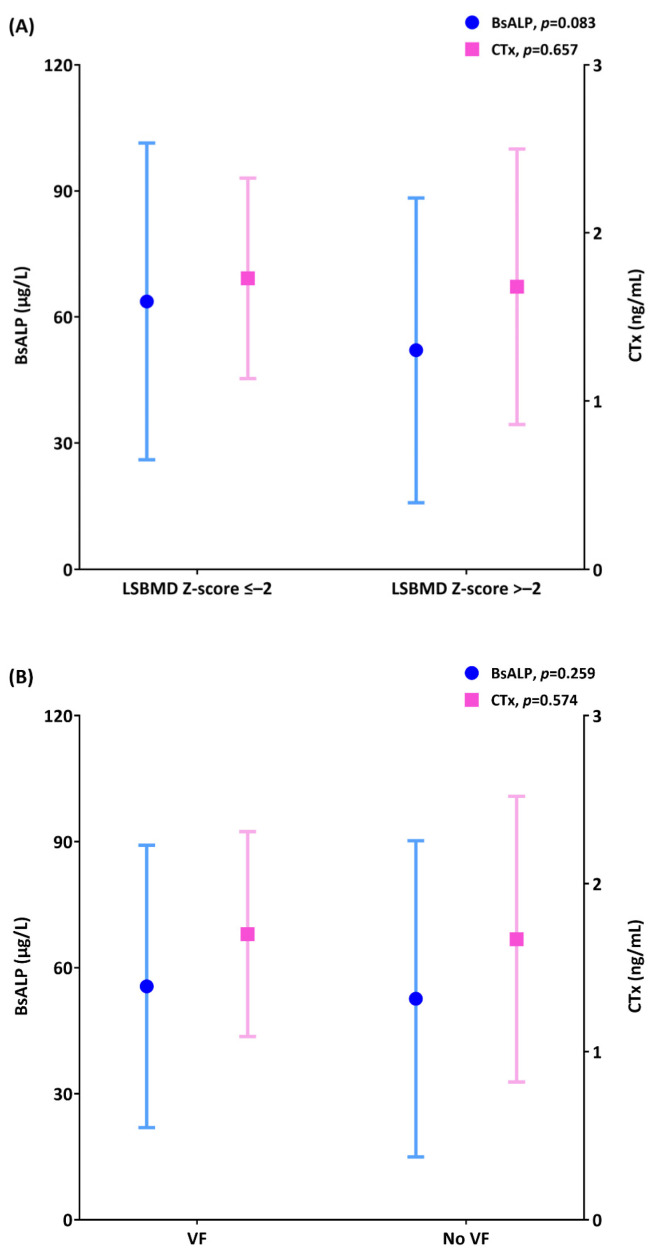
Bone-specific alkaline phosphatase (BsALP) and c-telopeptide of collagen type 1, β-crosslink (CTx) bars are drawn to compare their values according to the presence of (**A**) lumbar spine bone mineral density (LSBMD) Z-scores of negative −2.0 and (**B**) vertebral fractures (VFs). Circles and squares indicate BsALP and CTx, respectively, while each whisker indicates standard deviation.

When comparing two groups of patients, LSBMD Z-scores of ≤−2.0 and >−2.0, the values for BsALP were 63.6 ± 37.71 μg/L and 52.08 ± 36.23 μg/L, respectively, while for CTx they were 1.73 ± 0.59 ng/mL and 1.68 ± 0.82 ng/mL (Figure 1A). When comparing groups with and without vertebral fractures, the values for BsALP were 55.55 ± 33.61 μg/L and 52.6 ± 37.63 μg/L, respectively, while for CTx, they were 1.7 ± 0.61 ng/mL and 1.67 ± 0.85 ng/mL (Figure 1B). Both BsALP and CTx were greater in the groups with LSBMD Z-score of ≤−2.0 and with vertebral fracture; however, they were not statistically significant. For other bone markers such as calcium, phosphorus, 25OHD3, PTH, P1NP, BsALP to CTx ratio, and P1NP to CTx ratio, there were no significant differences between the groups.

### 3.3. Relationship Between BsALP and CTx with Other Bone Markers

When comparing the values of BsALP with other BTMs, calcium, phosphorus, and 25OHD3 were positively correlated with BsALP (*p* < 0.001), as well as with CTx. Meanwhile, PTH was not correlated with either BsALP or CTx (Table 2). Among different serum BTMs, calcium was the most closely correlated to BsALP (ρ = 0.497), while phosphorus was the most closely correlated to CTx (ρ = 0.492) (Table 2).

### 3.4. Association Between LSBMD and BTMs

In the univariate regression analysis, BMI Z-scores and LSBMD Z-scores were positively correlated (*p* < 0.001), while the age when the sampling was performed and other BTMs did not show significant correlations with LSBMD Z-scores (Table 3). In the multivariate regression analyses, BMI Z-scores and LSBMD Z-scores showed a positive correlation (*p* < 0.004) similar to the univariate regression, while the age when the sampling was performed showed a negative correlation (*p* = 0.032). In the multivariate regression analyses, BsALP and LSBMD Z-scores showed a negative correlation (*p* < 0.007). As with CTx, it seemed to be positively correlated with LSBMD Z-scores but was not statistically significant (Table 3). Although age, BMI Z-score, and BsALP all showed statistically significant correlations, either positive or negative, with the LSBMD Z-score, BsALP was most closely related with the smallest β (β = −0.011) (Table 3).

The logistic regression models showed that as the BMI Z-score decreased, the probability of LSBMD Z-score being ≤−2 increased with an OR of 0.763 (*p* = 0.024). After adjusting for underlying diseases and sex, as the BMI Z-score decreased and BsALP increased, the probability of LSBMD Z-score being ≤−2 increased with ORs of 0.515 and 1.043, respectively (*p* = 0.016, 0.048) (Table 4).

When comparing the BTMs with the vertebral fracture while the underlying diseases and sex were adjusted, as age and BsALP increased and 25OHD3 decreased, the probability of vertebral fracture increased with ORs of 1.597, 1.035, and 0.943, respectively (*p* = 0.019, 0.005, and 0.035). Age was most positively related to the vertebral fracture, followed by BsALP, and lastly 25OHD3.

The same comparison using a logistic regression model was performed with CTx. The multiple logistic regression showed that as the BMI Z-score decreased, the probability of LSBMD Z-score being ≤−2 increased with an OR of 0.437 (*p* = 0.007) after adjusting for underlying diseases and sex, similar to what was found with BsALP. However, the comparison with CTx with the LSBMD Z-score being ≤−2 and with the presence of vertebral fracture gave ORs of 0.419 and 1.001, respectively (*p* = 0.156, 0.997), which is not statistically significant (Supplementary Table S1).

A contingency table was performed to measure the difference between the frequency of LSBMD Z-score (0 or lower/−2 or lower) and the presence of vertebral fracture (yes/no). *χ*^2^ of LSBMD Z-score being ≤−2 for vertebral fracture was higher than the LSBMD Z-score being ≤0 (34.4 vs. 16.8; *p* < 0.001), indicating that the lower the LSBMD Z-score, the higher the risk of vertebral fracture (Supplementary Table S2).

## 4. Discussion

What is true for adults may not necessarily be true for children, and the use of BTMs in adults may not be the same in children. During puberty, skeletal development accelerates remarkably, and bones change their shape and dimensions during growth, a process called bone modeling [18]. BTMs change during growth as a function of age and sex; thus, comparing BTMs directly with LSBMD without any adjustments will not give any correlation in children. In this study, after adjusting for underlying disease and sex, BsALP and LSBMD Z-score being ≤−2 were positively correlated, as well as when comparing BsALP with the presence of vertebral fracture. Since the diagnosis of pediatric osteoporosis is based on LSBMD Z-score and the presence of vertebral fracture, the fact that both are related to BsALP is meaningful.

Serum BsALP is a specific and sensitive marker of bone formation, whereas CTx is a marker of bone resorption. Thus, biochemical bone markers, including BsALP and CTx, are useful tools for dynamic assessment of bone turnover, which, in association with more “static” densitometry measures obtained by DXA, provides a more complete evaluation of bone mass status [19]. Since measurements of biochemical bone markers can be repeated in short intervals, early detection of the effects of disease or treatments is possible, compared to DXA, as it needs longer intervals to reveal physical and densitometric changes in the bone.

There are previous studies about BTMs in the pediatric population with chronic diseases. Jackmann et al. [20] evaluated the vitamin D status and serum BTMs in children with hemato-oncologic diseases. The BTMs studied were serum PTH, BsALP, NTx, and CTx. The study showed that vitamin deficiency was observed in one-third of children with cancer, and the BTMs, both bone formation and bone resorption markers, were decreased in children with leukemia, compared to those who had solid tumors or bone marrow failure, possibly due to the suppression of osteoblasts and osteoclasts by leukemic cells [20]. Another study by Aksoy et al. [21] evaluated the relationship between BMD and BTMs in epileptic children, as antiepileptic drugs are known to affect bone metabolism. The BTMs studied included serum alkaline phosphatase (ALP) and CTx. BTMs were higher in children treated with carbamazepine (CBZ) than those who were either treated with valproic acid (VPA) or the healthy control group, who were not treated [21]. The study demonstrated that BTMs increased with CBZ and decreased with VPA treatment without affecting BMD and vitamin D levels in prepubertal epileptic children.

Due to the dynamic nature of BTMs, especially in children, there have been many attempts to find the reference ranges for BTMs in pediatric populations. Zhang et al. [22] conducted a study to establish the reference ranges of BTMs in southwest China. The authors pointed out that the BTM reference values established by other laboratories did not apply to children in southwest China, because they have a large span of altitude, and the ethnic groups are complex and diverse. Although they have defined reference ranges for BTMs including CTx and P1NP, they pointed out that they lack multicenter data and the differences between different regions and ethnicities have not been studied, so the application of the reference value needs further verification [22]. Further studies regarding the reference ranges of BTMs that encompass different ethnicities, geography, lifestyle, and epidemiological manifestations of disease and analytical methods used are warranted.

In adults, the relationship between BTMs and BMD was studied previously. Vasikaran et al. [23] suggested that BTMs may provide information on fracture risk; thus, fracture risk prediction might be enhanced by their inclusion in assessment algorithms [23]. Additionally, several studies have described a significant relationship between the reduction in BTMs following anti-resorptive therapy and the reduction in vertebral and non-vertebral fracture risk. Nakamura et al. [24] suggested that BsALP was negatively correlated with BMD, and that BsALP represented a useful serum marker to evaluate LSBMD during denosumab therapy in postmenopausal osteoporotic women [24]. When antiresorptive therapy was initiated in postmenopausal osteoporosis, resorption markers decreased first, followed by formation markers such as BsALP and P1NP [25]. Among different types of BTMs, CTx and P1NP showed the largest reduction in resorption and formation markers, respectively, and the magnitude of changes in each marker is different for different types of oral bisphosphonate used, which reflects the potency of the treatment [26].

Since CTx and P1NP are more widely studied in the adult population, a further study with CTx and P1NP in the pediatric population may better describe the relationship between CTx and P1NP with LSBMD and/or fracture risk. Additionally, unlike in adults, adjustment for age, not only for sex as conducted in this study, will be necessary for children, as it will give a better understanding of the relationship between BTMs and LSBMD. However, children are different from adults in that they undergo growth and rapid changes in bone modeling. The nature of BTMs might be different in children, and a different approach in children can give different results than in adults. In this study, we discovered the possible use of BsALP when predicting bone health in patients with pediatric osteoporosis.

This study had several limitations. First, it was carried out in a single tertiary center, resulting in a small sample size. Since the study was performed retrospectively, we were only able to include patients with underlying diseases with both LSBMD and BTM measurements. We did not have data for the healthy population to compare with. Second, the date BMD was measured was not necessarily the same day the BTMs were measured. Although we only included patients with less than 6 months interval between both measurements to reduce errors that might have resulted from the difference in time, the results might have been different if the BTM samples were taken the same day as LSBMD was measured. Especially, since vitamin D can show seasonal variability, due to the difference in sunlight exposure, considering the season when the BTMs was taken might have clarified the results. Third, although the reference values of BTMs in children are not established, the reference values are likely to differ according to age and sex, especially in the pediatric population. Although we adjusted for sex, we did not have enough sample size to adjust for age. Finally, the cross-sectional study design did not enable the comparison of the changes in BsALP and/or CTx, and a longitudinal study would be a better model.

## 5. Conclusions

Low bone mass, specifically when LSBMD Z-score is ≤−2 and with vertebral fracture, is positively correlated with BsALP after adjusting for underlying diseases and sex. This suggests the possible application of BsALP as a complementary tool to evaluate secondary osteoporosis in children and adolescents.

## Figures and Tables

**Table 1:** Clinical characteristics of the study participants.

	Total (*n* = 280)
**Males, *n* (%)**	95 (33.9)
**Age, years**	15.4 ± 2.07
**Anthropometry, Z-score**	
**Height**	−0.34 ± 1.22
**Weight**	−0.38 ± 1.43
**Body mass index**	−0.27 ± 1.54
**Underlying conditions, *n* (%)**	
**Hemato-oncologic**	172 (61.4)
**Rheumatic**	36 (12.9)
**Gastrointestinal**	35 (12.5)
**Endocrinologic**	15 (5.4)
**Miscellaneous**	22 (7.9)
**Age at diagnosis, years**	10.34 ± 4.34
**Bone mineral density**	
**Lumbar spine, z-score**	−0.52 ± 1.23
**Low bone mass, *n* (%)**	
**−1.0 < *n*** ≤ **0**	116 (41.4)
**−2.0 < *n*** ≤ **−1.0**	59 (21.1)
**−3.0 < *n*** ≤ **−2.0**	26 (9.3)
≤ **−3.0**	7 (2.5)
**Right femur, Z-score**	−1.03 ± 1.31
**Left femur, Z-score**	−1.07 ± 1.29
**Vertebral fractures**	79 (28.2)
**Bone turnover markers**	
**Bone-specific** **alkaline phosphatase,** **μg/L, median (IQR)**	44.3 (20.4, 80.4)
**CTx, ng/mL, median (IQR)**	1.62 (1.09, 2.25)
**P1NP, ng/mL, median (IQR)**	221 (107, 720)
**Calcium, mg/dL**	9.46 ± 0.40
**Phosphorus, mg/dL**	4.46 ± 0.65
**25 (OH) vitamin D total, ng/mL**	22.74 ± 11.64
**Parathyroid hormone-intact, pg/mL** **Bone-specific alkaline phosphatase to CTx ratio** **P1NP to CTx ratio**	40.47 ± 19.76 34.28 (20.99, 49.94) 176.62 (118.78, 358.02)

All values are expressed as mean ± standard deviation unless mentioned. CTx: C-telopeptide of collagen type 1, β-crosslink, P1NP: total procollagen 1 N-terminal propeptide.

**Table 2:** Correlations of bone-specific alkaline phosphatase and c-telopeptide of collagen type 1 with other bone markers.

	BsALP		CTx	
	ρ	*p*	ρ	*p*
Calcium	**0.497**	***<0.001***	**0.226**	***0.005***
Phosphorus	**0.444**	***<0.001***	**0.492**	***<0.001***
25OHD3	**0.284**	***<0.001***	**0.199**	***0.014***
PTH	−0.074	*0.12*	0.099	*0.227*

25OHD3, calcidiol; BsALP, bone-specific alkaline phosphatase; CTx, c-telopeptide of collagen type 1, β-crosslink; PTH, parathyroid hormone.

**Table 3:** Univariate and multivariate regression analyses of lumbar spine bone mineral density, demonstrating the effect of bone-specific alkaline phosphatase and c-telopeptide of collagen type 1, β-crosslink.

	LSBMD *Z*-Score					
	Univariate		Multivariate			
	β	*p*	β	*p*	β	*p*
Age	0.031	*0.381*	**−0.151**	***0.032***	*−0.057*	*0.357*
BMI *Z*-score	**0.285**	***<0.001***	**0.363**	***<0.004***	***0.361***	***<0.004***
Calcium	−0.346	*0.275*	0.021	*0.946*	*−0.123*	*0.699*
Phosphorus	−0.29	*0.133*	−0.365	*0.073*	***−0.558***	***0.013***
25OHD3	−0.014	*0.186*	0.007	*0.52*	*−0.005*	*0.658*
BsALP	−0.004	*0.056*	**−0.011**	***0.007***		
CTx	−0.236	*0.128*			*0.121*	*0.494*

25OHD3, calcidiol; BMI, body mass index; BsALP, bone-specific alkaline phosphatase; CTx, c-telopeptide of collagen type 1, β-crosslink; LSBMD, lumbar spine bone mineral density.

**Table 4:** Logistic regression models demonstrating the effect of bone-specific alkaline phosphatase on low bone mass and vertebral fractures.

	LSBMD *Z*-Score ≤−2				VF			
	Univariate		^†^ Multivariate		Univariate		^†^ Multivariate	
	OR	*p*	OR	*p*	OR	*p*	OR	*p*
Age	1.07	*0.421*	1.727	*0.081*	1.037	*0.572*	**1.597**	***0.019***
BMI *Z*-score	**0.763**	***0.024***	**0.515**	***0.016***	0.852	*0.069*	0.872	*0.409*
Calcium	2.68	*0.229*	2.097	*0.534*	1.247	*0.702*	0.785	*0.753*
Phosphorus	1.248	*0.629*	0.707	*0.703*	1.347	*0.433*	1.34	*0.615*
25OHD3	1.006	*0.781*	0.943	*0.14*	0.978	*0.257*	**0.943**	***0.035***
BsALP	1.008	*0.073*	**1.043**	***0.048***	1.002	*0.543*	**1.035**	***0.005***

^†^ Adjustment for the underlying disease and sex included. 25OHD3, calcidiol; BMI, body mass index; BsALP, bone-specific alkaline phosphatase; LSBMD, lumbar spine bone mineral density; VFs, vertebral fractures.

## Data Availability

All the data analyzed in this study are not publicly available for the privacy of the research participants but are available from the corresponding author (M.B.A.) upon reasonable request.

## Supplementary Materials

The following supporting information can be downloaded at: https://www.mdpi.com/article/10.3390/diagnostics15050630/s1, Table S1: Multiple logistic regression models demonstrating the effect of c-telopeptide of collagen type 1, β-crosslink, and bone-specific alkaline phosphatase on low bone mass and vertebral fractures; Table S2: Correlations between the prevalence of vertebral fractures and the degree of low bone mass.
