# Supplementary material for: Bone-Specific Alkaline Phosphatase as a Complementary Diagnostic Marker for the Assessment of Children and Adolescents with Secondary Osteoporosis
Source: Diagnostics (Basel). 2025 Mar 5;15(5):630. doi: 10.3390/diagnostics15050630 (PMC11898864; doi:10.3390/diagnostics15050630)
Supplement: Supplementary file 1 [file diagnostics-15-00630-s001.zip › diagnostics-3441802-supplementary.pdf]

Supplementary Files

Table S1. Multiple logistic regression models demonstrating the effect of c-telopeptide of collagen type 1, β-crosslink, and bone-specific alkaline phosphatase on low bone mass and vertebral fractures

	LSBMD *Z*-score ≤-2		VF	
	†Multivariate			
	**OR**	***p***	**OR**	***p***
Age	1.107	*0.613*	1.161	*0.301*
BMI *Z*-score	**0.437**	***0.007***	0.845	*0.325*
Calcium	1.308	*0.824*	0.789	*0.747*
Phosphorus	4.703	*0.068*	2.084	*0.176*
25OHD3	1.004	*0.914*	0.975	*0.285*
CTx	0.419	*0.156*	1.001	*0.997*

†Adjustment for the underlying disease and sex included
25OHD3, calcidiol; BMI, body mass index; CTx, c-telopeptide of collagen type 1, β-crosslink; LSBMD, lumbar spine bone mineral density; VF, vertebral fractures

Table S2. Correlations between the prevalence of vertebral fractures and the degree of low bone mass

	LSBMD *Z*-score					
	≤0 (*n*=172)			≤-2 (*n*=26)		
VF	**n(%)**	**χ^2**	***p***	**n(%)**	**χ^2**	***p***
	**64(37.2)**	**16.8**	***<0.001***	**26(68.4)**	***34.4***	***<0.001***

LSBMD, lumbar spine bone mineral density; VF, vertebral fracture
